# Land System Science: between global challenges and local realities^☆^

**DOI:** 10.1016/j.cosust.2013.08.001

**Published:** 2013-10

**Authors:** Peter H Verburg, Karl-Heinz Erb, Ole Mertz, Giovana Espindola

**Affiliations:** 1 Institute for Environmental Studies, VU University, Amsterdam, The Netherlands; 2 Institute for Social Ecology, Alpen-Adria University, Klagenfurt/Vienna/Graz, Austria; 3 Department of Geosciences and Natural Resource Management, University of Copenhagen, Denmark; 4 Brazilian National Institute for Space Research, Brazil

## Abstract

This issue of *Current Opinion in Environmental Sustainability* provides an overview of recent advances in Land System Science while at the same time setting the research agenda for the Land System Science community. Land System Science is not just representing land system changes as either a driver or a consequence of global environmental change. Land systems also offer solutions to global change through adaptation and mitigation and can play a key role in achieving a sustainable future earth. The special issue assembles 14 articles that entail different perspectives on land systems and their dynamics, synthesizing current knowledge, highlighting currently under-researched topics, exploring scientific frontiers and suggesting ways ahead, integrating a plethora of scientific disciplines.

**Current Opinion in Environmental Sustainability** 2013, **5**:433–437

For a complete overview see the Issue

1877-3435/$ – see front matter, © 2013 The Authors. Published by Elsevier B.V. All rights reserved.

**http://dx.doi.org/10.1016/j.cosust.2013.08.001**

## Land System Science

Changes in land systems, human-induced transformations of ecosystems and landscapes and the resulting changes in land cover, reach far beyond local alterations and are pervasive factors of global environmental change. Today, more than 75% of the Earth's ice-free land shows significant evidence of land use induced alterations of many environmental processes, such as primary production, the water cycle, biogeochemical cycles, the climate system, and biodiversity. On the other hand, land provides vital socioeconomic resources to society, such as of food, fuel, fibres and many other ecosystem services that support production functions, regulate risks of natural hazards, or provide cultural and spiritual services. Land system changes are the direct result of human decision making at multiple scales, with far reaching consequences for the Earth System, that feedback on human well-being and decision making. Thus, land system change is both a cause and consequence of socio-ecological processes that encompasses a huge range of spatio-temporal scales.

Land systems represent the terrestrial component of the Earth system and encompass all processes and activities related to the human use of land, including socioeconomic, technological and organizational investments and arrangements, as well as the benefits gained from land and the unintended social and ecological outcomes of societal activities. Thus, Land System Science has emerged to serve as a platform for integration of these different dimensions of global environmental change research, and aims at offering potential options for mitigation and adaption to environmental change, for example through modified land system architecture [1]. By studying the mutual interplay between social and ecological systems that shape land use and land cover, land system science operates at the interface of the social and natural sciences and requires a high level of interdisciplinary collaboration across academic disciplines as is reflected in the contributions to this issue.

Land system science has developed over the past twenty years from the study of Land Use and Land Cover Change, which initially was dominated by monitoring and modelling of the ecological impacts of land cover changes such as deforestation and desertification on the natural system [2, 3, 4]. Gradually, the research field has become more integrative, focusing on both the drivers and impacts of land change as part of global environmental change. The growing group of researchers engaged in this field led to the emergence of ‘Land Change Science’ as a separate, interdisciplinary, research field engaging scientists across the social, economic, geographical and natural sciences [5, 6]. The increasing attention for feedbacks between drivers and impacts including adaptive behaviour [7], the interactions between social and ecological systems and teleconnections between world regions [8, 9] and between cities and their rural hinterlands [10] have motivated an integrated socio-ecological systems perspective. In this perception, land systems are acknowledged as resulting from the dynamic interactions within the socio-ecological system. This perspective has also moved land system science from a focus on the most dramatic land cover changes to giving more attention to subtle changes of human interactions with the natural surroundings, including land management and the provisioning of a wide range of ecosystem services. The articles in this issue strongly reflect this shift in perspective by explicitly addressing changes in land management and the modes in which land is governed.

The Land System Science community is organized within the Global Land Project, one of the core projects of both the International Geosphere Biosphere Programme (IGBP) and the International Human Dimensions Programme on Global Environmental Change (IHDP) commissioned by the International Council for Science (ICSU) and the International Social Science Council (ISSC). In 2013, a new programme gathering all previous global environmental change programmes was established and named ‘Future Earth’ in response to a visioning process on Earth System Science and Global Sustainability initiated by ICSU [11]. The aims of the new programme, that will also host the Global Land Project, include a stronger interdisciplinary approach and a stronger focus on science that supports sustainability transitions through co-design and co-production of research together with important stakeholders. While interdisciplinarity is in the genes of Land System Science, a stronger engagement in the development of sustainability solutions provides an important opportunity for the researchers engaged in this field. Traditionally Land System Science is closely related to the fields of land use planning and land use policy. However, scientific insights are not always easily integrated in these processes and much land is owned and managed by private land owners that are not always responsive to planning and policy. Therefore, new ways of linking science and practice need to be developed to effectively translate scientific findings into sustainability solutions and implementation in practice. Important ways forward in this perspective include the evaluation and design of alternative ways to govern land resources [12, 13, 14] and the use of land systems architecture in the design of novel land systems that more optimally use spatial and temporal interactions within the land system configuration to provide ecosystem services and adaptive capacity under conditions of global environmental change [1, 15, 16]. Many of the articles in this issue address these challenges and illustrate the role of Land System Science as a central and critical component of global sustainability science.

## Synthesis of Land System Science

The articles in this issue provide a synthesis of many, interrelated, topics in Land System Science, either from a thematic or methodological perspective. Overall, the articles can be divided into four main approaches to studying land systems: land systems dynamics, land use intensity, impacts of land change and governance of land systems.

The first four articles deal with linkages between the local and global, teleconnections and modelling approaches to understanding and influencing land system dynamics. Meyfroidt *et al.* [17] use the concept of telecoupling to review how local processes of land system change are affecting other, often distant, regions and how demand and policy often have indirect consequences in different world regions. The authors argue for combining place-based research and global modelling to better understand the causal links between flows of goods and services and land system change. Güneralp *et al.* [18] state that land systems research should address the interactions between urban and rural areas as well as the transformations within urban and peri-urban landscapes. They propose a conceptual framework that allows identifying and examining linkages between urbanization dynamics and the associated land system changes, which can occur in distant places, and discuss the sustainability implications of this increasingly important land system change.

Brown *et al.* [19] discuss the ways in which different model types can help in understanding future land system dynamics and inform the formulation, implementation and monitoring of land use policy and planning processes. From a review of current models they conclude that a majority of models are focusing on exploring socio-ecological system function, scenario analysis and ex-ante assessment. Seppelt *et al.* [16] argue for a complementary use of exploratory modelling and optimization approaches to explore the options of land system configurations that best meet the goods and services demands of society. While such combinations of modelling concepts have been proposed before [20], little operational examples are yet available and the combined use of such approaches in co-designing land use planning and policy with stakeholders has not yet been fully explored.

Four articles specifically deal with the intensity and extent of land systems. Erb *et al.* [21] provide a conceptual framework for studying land use intensity based on a review of theoretical concepts and indicators available in the literature. They indicate that the integration of three dimensions of land-use intensity is needed, combining metrics for inputs and outputs of land based production systems as well as system parameters that can reflect the unintended outcomes of land use and represent powerful drivers of land system dynamics. Such structured analysis of land use intensity is rarely conducted in the current Land System Science literature. Munroe *et al.* [22] address the extensification processes taking place in several regions around the world and argue that agricultural abandonment does not always result in a straightforward trajectory of forest recovery. Agricultural abandonment is the starting point of many alternative pathways of changes in land management, vegetation and the use and governance of the natural resources. The different trajectories are strongly dependent on the local socio-economic and biophysical context, and impacts may have strong tradeoffs between ecosystem services provided by the changing land system.

Such tradeoffs are also key to the article of Grau *et al.* [23] who discuss alternative land development trajectories of land sparing and land sharing, that is, a separation of natural area conservation from intensive production systems versus a more multi-functional integration of service provisioning on the same area. While some of these discussions are dominated by strong generalizations, the article in this issue pleas for a more nuanced analysis, the integration of different development trajectories and a broadening of the scale of analysis while accounting for global teleconnections. Key to such analysis is an improved understanding of land use intensity, and the consideration of local socioeconomic and ecological constraints. Finally, Kuemmerle *et al.* [24] discuss strengths and weaknesses of remote sensing as provider of data on land cover change. Many of the more subtle changes in land use that occur within a certain land cover type, such as changes in land-use intensity, management and spatial organization, cannot be straightforwardly derived from remote sensing. Hence, different methods and approaches need to be followed to acquire relevant information on these changes. In their review of the state of the art in land-use intensity monitoring, they outline major challenges, but also opportunities emerging from the availability of new technologies, for mapping across different land-use types.

The impacts — and feedbacks — of changes in land use intensity and land cover extent are addressed by the next group of four articles. Verburg *et al.* [25] discuss how the land system plays a crucial role within the food system, dependent on the connections between local food production systems and regional to global markets. In their article they sketch a land systems approach for contributing to sustainable local food production systems in the context of a globalized food system. Change in land systems also affects natural systems. Nagendra *et al.* [26] outline how changes and modifications in land cover constitute the most dominant drivers of biodiversity loss globally. These changes affect the structure and function of ecosystems and alter their capacity to provide sustained ecosystem services for human well-being. Moreover, there are challenges and critical gaps in our knowledge on the links between biodiversity, ecosystem function and ecosystem services. The authors suggest that research on ecosystem services needs to expand beyond its largely descriptive approach to a deeper understanding of socio-ecological functions. Crossmann and colleagues [27] discuss how land system science can contribute to mainstreaming and operationalizing the ecosystem service concept in land management and policy. It is argued that a further integration between socio-economic and biophysical approaches in ecosystem service assessments and land governance analysis is needed to support the development and implementation of more sustainable land management practices.

While the impact of land system change on food systems, biodiversity, ecosystem services has been discussed frequently, the relationships between land system changes and human health issues have been given much less attention, especially due to the very different disciplinary communities addressing these issues. Messina *et al.* [28] review the literature in this field and discuss the ways in which knowledge from both fields can complement each other and how problems due to the different ontologies of the different disciplines can be overcome.

Two articles provide more specific insight on land governance issues. Sikor *et al.* [14] present an overview of alternative ways of governance of land resources and argue that land is now governed more on the basis of flows of resources or goods rather than on territorial arrangements. They conclude that this has generated new forms of social exclusion and inequity, and that future land policy needs to combine territorial and flow-centered arrangements. Messerli *et al.* [29] focus on the processes of large scale land acquisitions that have received a lot of media attention and an increasing interest from the scientific community. The impacts of such large scale land acquisition on social systems, the environmental system and the food system are still largely unknown and very variable depending on local context and the mode of acquisition and governance. However, the scale of these acquisitions is very large and spread across a large part of (mainly) the developing world. Land acquisitions are one of the typically examples of how local realities of changes in land systems are intricately linked to the global challenge of sustainable and equitable development.

Finally, we acknowledge that a number of important aspects of Land System Science are not discussed in this issue — either because reviews of those topics have been published recently or because contributions were unavailable or not yet ready for publication. Examples of recent reviews on other important aspects of Land System Science include those on long term historic changes in land use [30], land use and the climate system [31, 32], forest transitions [33], land use data [34] and livestock systems [35]. Also, a series of meta-analysis of case studies on the drivers [36, 37, 38, 39, 40, 41] and consequences of land system change [42, 43] provide a useful synthesis of commonalities and context dependencies across place-based land systems research.

The synthesis of land change, through review, meta-analysis and the development of conceptual frameworks and theory, helps to bring together findings on land system changes across different scales and from different disciplinary perspectives. All papers in this issue identify new challenges based on the knowledge gaps identified within the review. This way, synthesis of current knowledge translates into an updated research agenda for the Land System Science community.
